# Angiopreventive Efficacy of Pure Flavonolignans from Milk Thistle Extract against Prostate Cancer: Targeting VEGF-VEGFR Signaling

**DOI:** 10.1371/journal.pone.0034630

**Published:** 2012-04-13

**Authors:** Gagan Deep, Subhash Chander Gangar, Subapriya Rajamanickam, Komal Raina, Mallikarjuna Gu, Chapla Agarwal, Nicholas H. Oberlies, Rajesh Agarwal

**Affiliations:** 1 Skaggs School of Pharmacy and Pharmaceutical Sciences, University of Colorado Denver, Aurora, Colorado, United States of America; 2 University of Colorado Cancer Center, University of Colorado Denver, Aurora, Colorado, United States of America; 3 Department of Chemistry and Biochemistry, University of North Carolina at Greensboro, Greensboro, North Carolina, United States of America; University of Nebraska Medical Center, United States of America

## Abstract

The role of neo-angiogenesis in prostate cancer (PCA) growth and metastasis is well established, but the development of effective and non-toxic pharmacological inhibitors of angiogenesis remains an unaccomplished goal. In this regard, targeting aberrant angiogenesis through non-toxic phytochemicals could be an attractive angiopreventive strategy against PCA. The rationale of the present study was to compare the anti-angiogenic potential of four pure diastereoisomeric flavonolignans, namely silybin A, silybin B, isosilybin A and isosilybin B, which we established previously as biologically active constituents in Milk Thistle extract. Results showed that oral feeding of these flavonolignans (50 and 100 mg/kg body weight) effectively inhibit the growth of advanced human PCA DU145 xenografts. Immunohistochemical analyses revealed that these flavonolignans inhibit tumor angiogenesis biomarkers (CD31 and nestin) and signaling molecules regulating angiogenesis (VEGF, VEGFR1, VEGFR2, phospho-Akt and HIF-1α) without adversely affecting the vessel-count in normal tissues (liver, lung, and kidney) of tumor bearing mice. These flavonolignans also inhibited the microvessel sprouting from mouse dorsal aortas *ex vivo*, and the VEGF-induced cell proliferation, capillary-like tube formation and invasiveness of human umbilical vein endothelial cells (HUVEC) *in vitro*. Further studies in HUVEC showed that these diastereoisomers target cell cycle, apoptosis and VEGF-induced signaling cascade. Three dimensional growth assay as well as co-culture invasion and *in vitro* angiogenesis studies (with HUVEC and DU145 cells) suggested the differential effectiveness of the diastereoisomers toward PCA and endothelial cells. Overall, these studies elucidated the comparative anti-angiogenic efficacy of pure flavonolignans from Milk Thistle and suggest their usefulness in PCA angioprevention.

## Introduction

Prostate cancer (PCA) is the most frequently diagnosed non-cutaneous malignancy among men in the United States, and is the second leading cause of cancer-related deaths [1]. Clinical and experimental evidence have suggested that human tumors could persist for years as microscopic lesions in a state of dormancy and their further growth is critically dependent upon attaining an ‘angiogenic phenotype’ [2], [3], [4], [5]. ‘Angiogenic switches’ involving the high VEGF and VEGF receptor (VEGFR) levels have been identified and considered responsible for PCA progression from low grade PIN (prostatic intraepithelial neoplasia) stage to high grade PIN and further to more aggressive, poorly differentiated, and androgen-independent malignant stages [6]. Furthermore, angiogenesis level in PCA has been correlated directly with Gleason score, tumor stage, progression, metastasis and survival [6], [7], [8]. Therefore, targeting angiogenesis has been the subject of several clinical investigations to improve the quality of life of cancer patients [9], [10], [11]. Furthermore, preventing the onset of angiogenesis in indolent tumors (referred as ‘angioprevention’) has been suggested as a novel and rationale approach to control PCA growth, malignant progression and metastasis to secondary sites.

**Figure 1:**
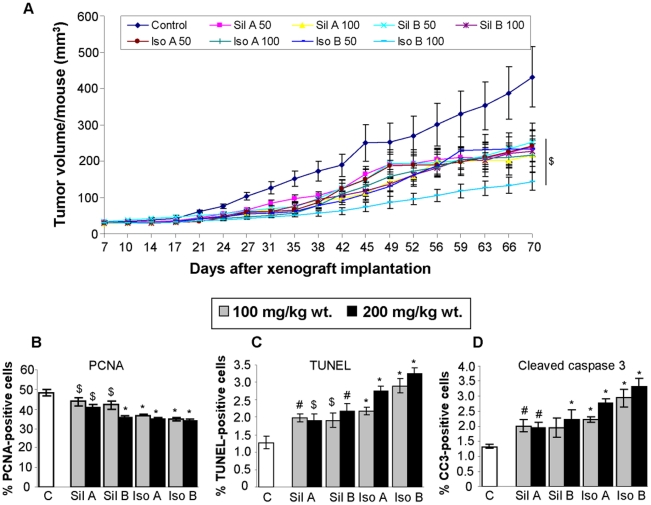
Flavonolignans inhibit PCA DU145 xenograft growth through targeting proliferation and apoptosis. DU145 xenografts were initiated and mice were administered either vehicle (CMC) or 50 and 100 mg/kg body weight doses of each diastereoisomer. (**A**) Tumor volume was measured and plotted as a function of time (days). Each value in the curves is mean ± SEM of 10–12 mice. (**B–D**) Xenograft tissues were analyzed for PCNA, TUNEL and cleaved caspase-3 (CC3) by IHC. The data shown in the bar diagrams is the mean± SEM of 4–5 samples. Abbreviations: Sil A: Silybin A; Sil B: Silybin B; Iso A: Isosilybin A, Iso B: Isosilybin B; *, p ≤ 0.001; #, p ≤ 0.01; $, p ≤ 0.05.

**Figure 2:**
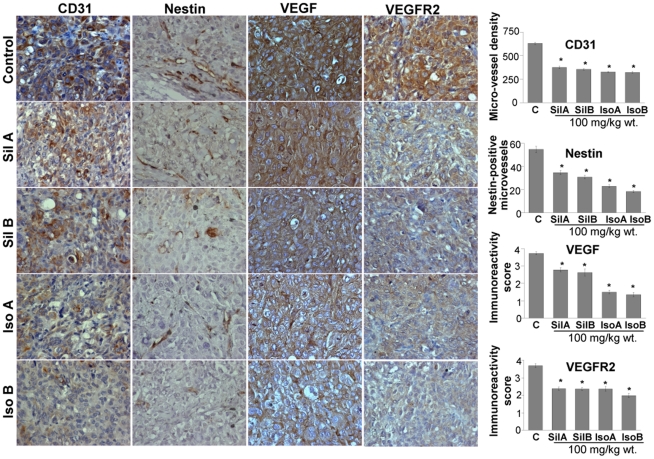
Flavonolignans inhibit angiogenesis *in vivo*. DU145 xenograft tissues were analyzed for CD31, nestin, VEGF and VEGFR2 by IHC. Quantitative analyses were performed using Zeiss Axioscope 2 microscope (Carl Zeiss, Germany) and photographs were originally captured (at 400x) with a Carl Zeiss AxioCam MrC5 camera with Axiovision Rel 4.5 software. The data shown in the bar diagrams is the mean ± SEM of 4–5 samples. Abbreviations: Sil A: Silybin A; Sil B: Silybin B; Iso A: Isosilybin A, Iso B: Isosilybin B; *, p ≤ 0.001.

**Figure 3:**
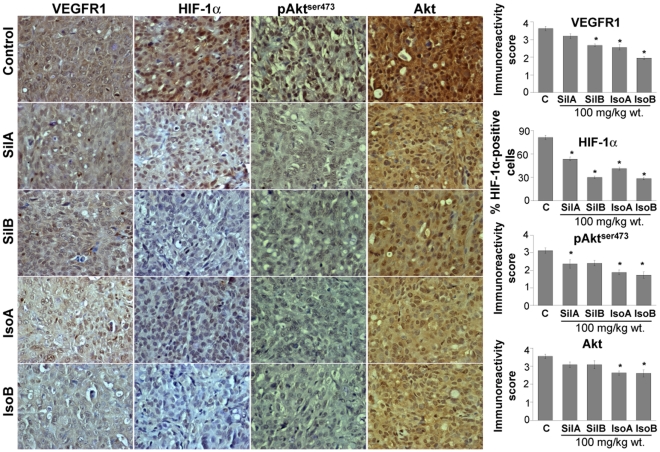
Flavonolignans decrease VEGFR1, HIF-1α, phosphorylated and total Akt levels in DU145 xenografts. DU145 xenograft tissues were analyzed for VEGFR1, HIF-1α, phosphorylated Akt^ser473^ and total Akt levels by IHC. Quantitative analyses were performed using Zeiss Axioscope 2 microscope (Carl Zeiss, Germany) and photographs were originally captured (at 400x) with a Carl Zeiss AxioCam MrC5 camera with Axiovision Rel 4.5 software. The data shown in the bar diagrams is the mean ± SEM of 4–5 samples. Abbreviations: Sil A: Silybin A; Sil B: Silybin B; Iso A: Isosilybin A, Iso B: Isosilybin B; *, p ≤ 0.001.

About four decades ago, Judah Folkman first predicted the potential role for anti-angiogenic inhibitors against solid cancers, and to date, several angiogenesis inhibitors have been tested against many malignancies [12], [13], [14], [15]. Many of these inhibitors have already been FDA approved for their use either alone or in combination with cancer chemotherapeutic drugs [13], [15], [16]. For example, humanized VEGF antibody was approved against colorectal, brain, lung, and renal cancers [16]. Similarly, the tyrosine kinase inhibitors sorafenib and sunitinib, which target VEGFR activity, have been approved for use against advanced renal cell carcinoma [16]. Although inhibiting angiogenesis in cancer, in principle, is a sound preventive/therapeutic strategy, the current approach of targeting a single molecule such as VEGF or VEGFR is flawed, as cancer cells develop resistance through circumventing these molecules and continue to spread vascular networks [17]. Beside their limited efficacy in terms of improvement in patient’s survival, these treatment options are extremely expensive and have shown unacceptable levels of toxicity [18], [19]. Therefore, we rationalized the need to identify non-toxic natural agents with broad spectrum anti-angiogenic efficacy; and in this regard, in the present study, we focused on the anti-angiogenic efficacy of four pure diastereoisomers from Milk Thistle (*Silybum marianum*) extract, namely silybin A, silybin B, isosilybin A and isosilybin B. These diastereoisomers are flavonolignans with an identical flavonoid moiety and differ only in their configurations about the lignan moiety, and their chemical and biological properties have been detailed earlier [20], [21], [22], [23], [24]. Here, for the first time we analyzed the angiopreventive efficacy of these flavonolignans in PCA xenograft model and various *ex vivo*, *in vivo* and *in vitro* angiogenesis assays.

**Figure 4:**
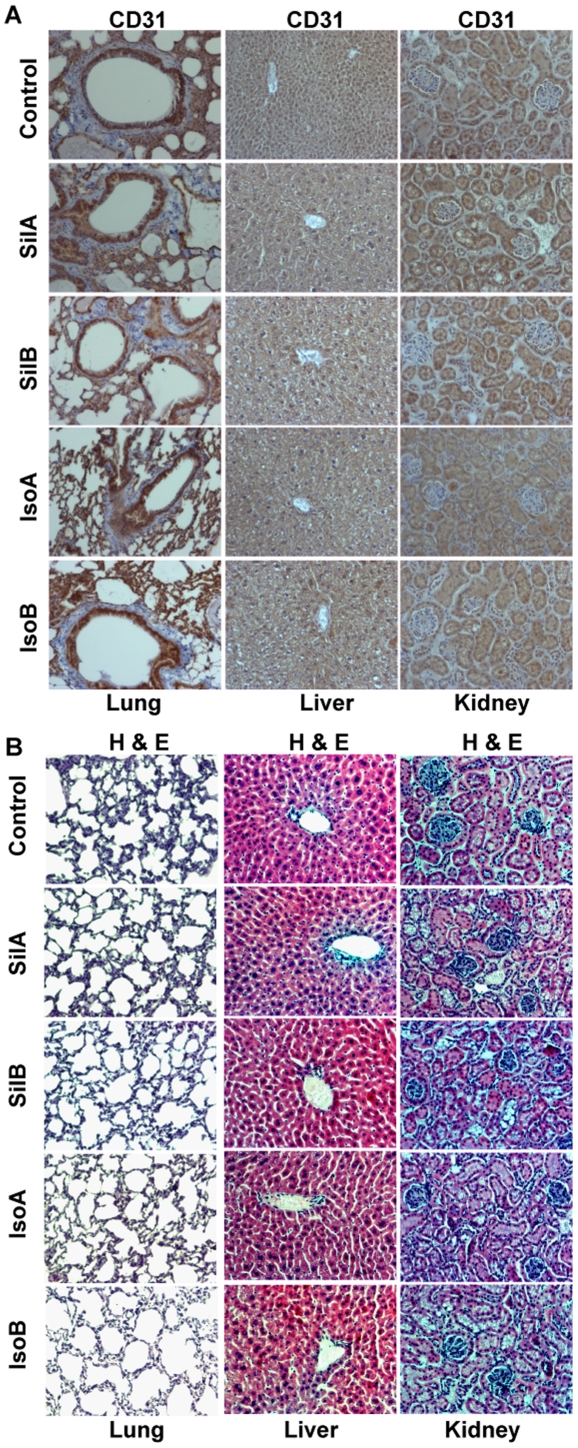
Feeding of pure flavonolignans did not affect angiogenesis and normal histology in non-target organs. (**A–B**) Lungs, liver and kidneys from each mouse were collected and analyzed for CD31 immunoreactivity as well as for histopathological analyses. Quantitative analyses were performed using Zeiss Axioscope 2 microscope (Carl Zeiss, Germany) and photographs were originally captured (at 400x) with a Carl Zeiss AxioCam MrC5 camera with Axiovision Rel 4.5 software. Abbreviations: Sil A: Silybin A; Sil B: Silybin B; Iso A: Isosilybin A, Iso B: Isosilybin B.

**Figure 5:**
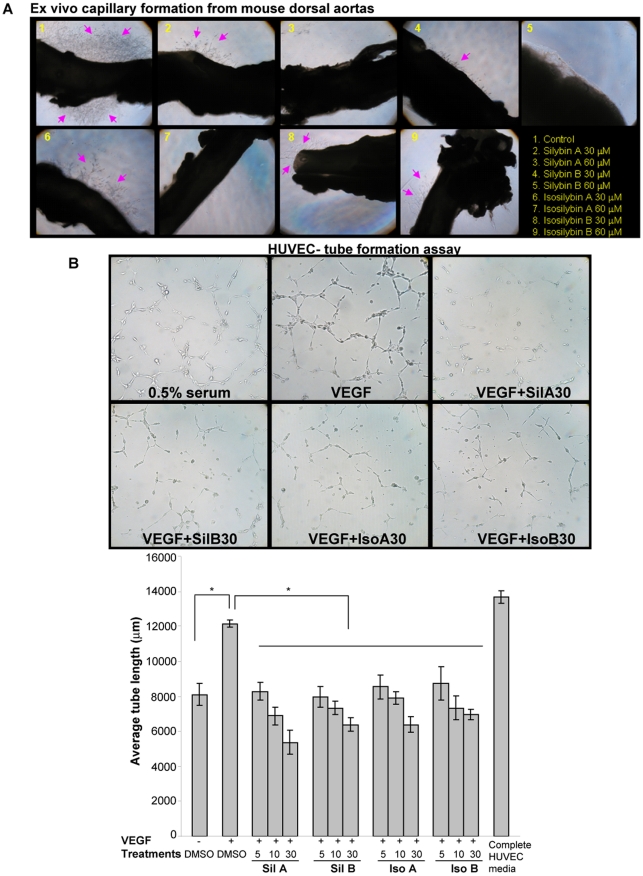
Flavonolignans inhibit angiogenesis in *ex vivo* and *in vitro* models. (**A**) **Flavonolignans inhibit angiogenesis** ***ex vivo*** **.** Mouse aortas were plated on matrigel and treated with flavonolignans. The arrows in the picture mark the emerging vessels from the aortas. (**B**) **Flavonolignans inhibit VEGF-induced tube formation in HUVEC.** HUVEC were plated on matrigel and effect of diastereoisomers treatment on VEGF-induced tube formation was analyzed. Representative tubular network photomicrographs are shown at 100x (top panel). Tube length was quantified as detailed in ‘Methods’ (bottom panel). Abbreviations: Sil A: Silybin A; Sil B: Silybin B; Iso A: Isosilybin A, Iso B: Isosilybin B; *, p ≤ 0.001.

**Figure 6:**
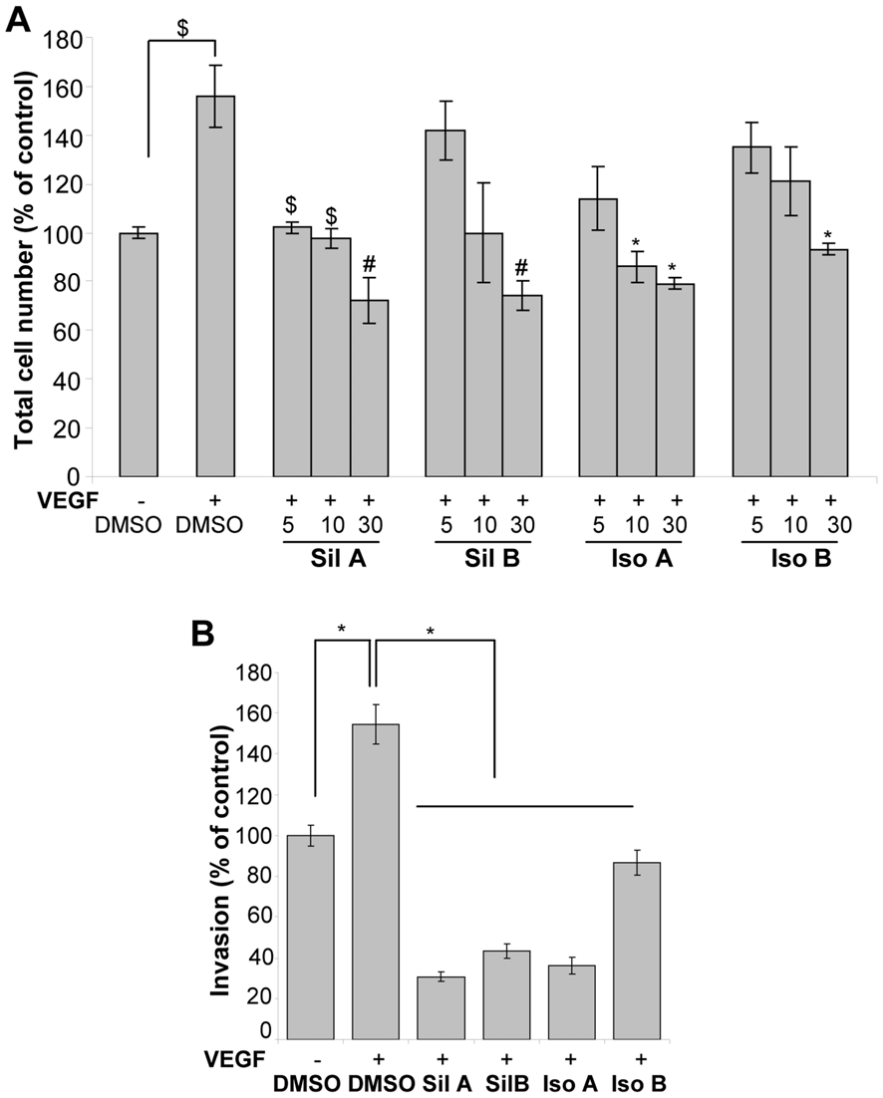
Flavonolignans inhibit VEGF-induced proliferation and invasion in HUVEC. (**A**) HUVEC were induced with VEGF and treated with each flavonolignan in 0.5% serum media, and total cell number was analyzed after 24 h. (**B**) HUVEC were plated in the upper chamber with DMSO or individual diastereoisomer, while VEGF was added in the lower chamber and HUVEC invasion was studied. Abbreviations: Sil A: Silybin A; Sil B: Silybin B; Iso A: Isosilybin A, Iso B: Isosilybin B; *, p ≤ 0.001; #, p ≤ 0.01; $, p ≤ 0.05.

**Figure 7:**
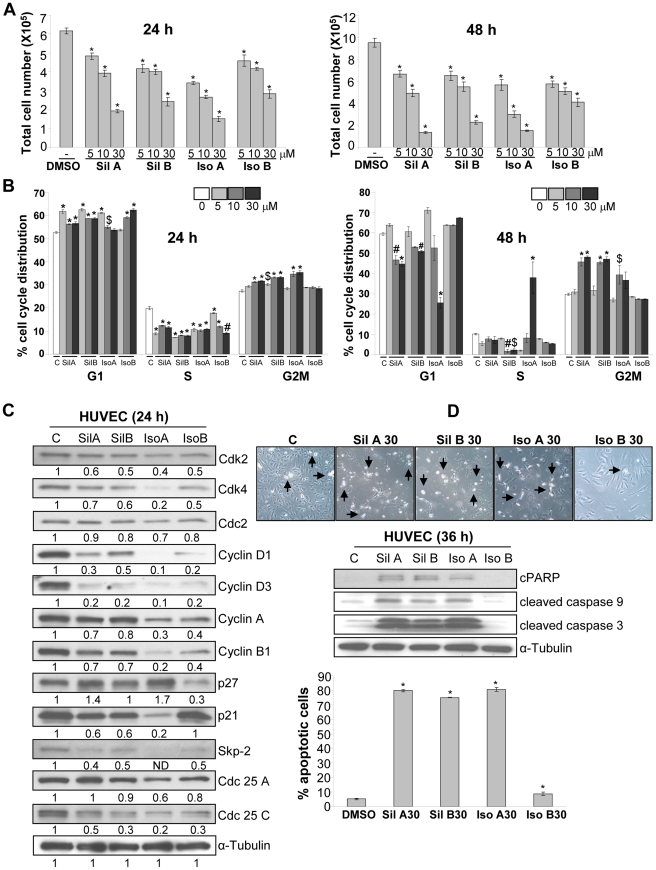
Effect of flavonolignans on viability, cell cycle distribution and apoptosis in HUVEC. (**A–B**) HUVEC were treated with DMSO or individual flavonolignan and analyzed for total cell number and cell cycle distribution. (**C**) HUVEC were treated with flavonolignans, and 24 h later, total cell lysates were prepared and analyzed for cell cycle regulators. The densitometry values presented below the bands are ‘fold change’ compared to control after loading control (α-tubulin) normalization. (**D**) HUVEC were treated with flavonolignans (at 30 µM dose) for 36 h and analyzed for morphology (representative photomicrographs are shown at 100x), levels of cPARP, cleaved caspase 3 and 9, and percentage apoptotic cells. Abbreviations: Sil A: Silybin A; Sil B: Silybin B; Iso A: Isosilybin A, Iso B: Isosilybin B; *, p ≤ 0.001; #, p ≤ 0.01; $, p ≤ 0.05.

**Figure 8:**
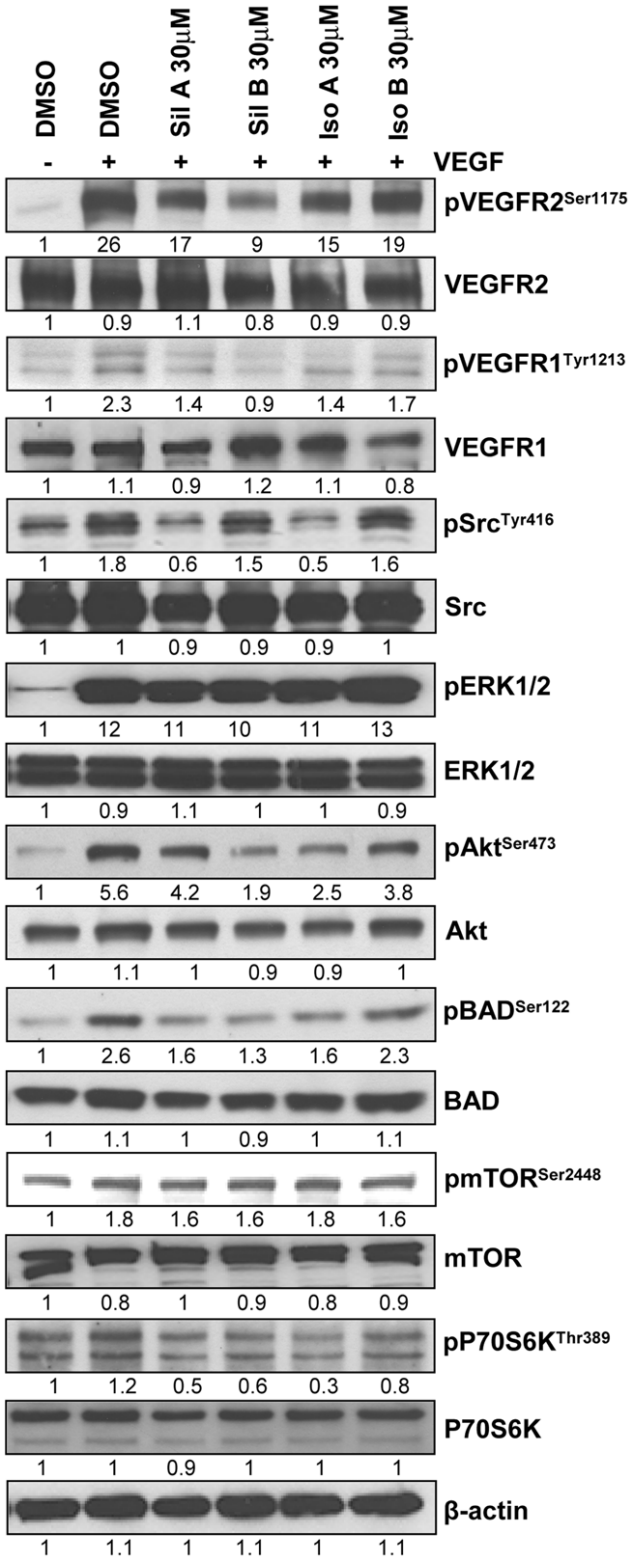
Effect of flavonolignans on VEGF-induced signaling cascade in HUVEC. HUVEC were serum starved for 22 h, treated with diastereoisomers for 2 h and stimulated with VEGF (10 ng/ml) for 10 minutes. Total cell lysates were prepared and analyzed for mentioned signaling molecules. The densitometry values presented below the bands are ‘fold change’ compared to control after loading control (β-actin) normalization.

**Figure 9:**
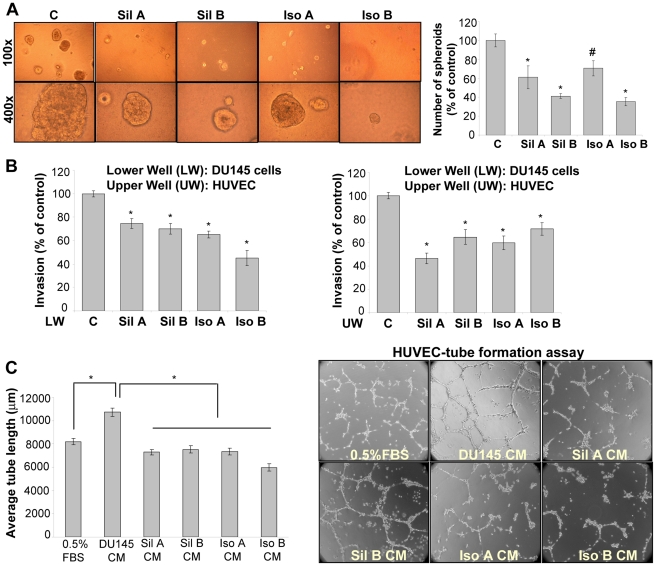
Differential effect of flavonolignans on human PCA DU145 cells and endothelial HUVEC in cell culture assays. (**A**) Effect of flavonolignans (at 90 µM dose) on the three dimensional growth of DU145 cells was studied as detailed in ‘Materials and Methods’. Representative spheroids photomicrographs are shown at 100x and 400x. (**B**) In Transwell invasion assay, either DU145 cells (plated in the lower chamber) or HUVEC (plated in the upper chamber) were treated with individual diastereoisomers (at 30 µM dose), and HUVEC invasiveness was measured. (**C**) DU145 cells were treated with individual diastereoisomers (at 30 µM dose) and conditioned media was collected. HUVEC were plated on matrigel along with 0.5% FBS or conditioned media from different treatment groups; and tube formation was analyzed. Representative photomicrographs of tubular network are shown at 100x. Abbreviations: Sil A: Silybin A; Sil B: Silybin B; Iso A: Isosilybin A, Iso B: Isosilybin B; CM: Conditioned media; *, p ≤ 0.001; #, p ≤ 0.01.

**Figure 10:**
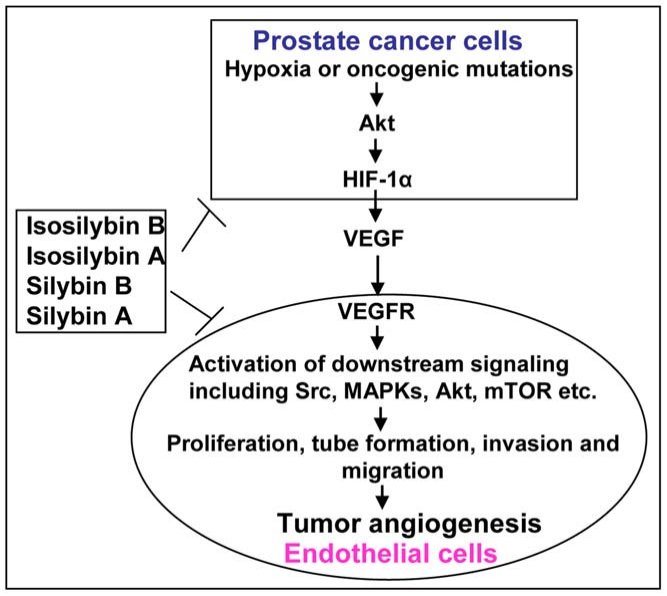
Diastereoisomers exhibit anti-angiogenic effects through targeting signaling molecules in both prostate cancer cells and endothelial cells. Silybin A, silybin B, isosilybin A and isosilybin B target angiogenesis in prostate tumors through targeting signaling molecules in PCA cells as well as in endothelial cells, the important component of PCA microenvironment.

## Methods

### Cell Line and Reagents

Human PCA DU145 cells were from ATCC (Manassas, VA) and cultured as described earlier [25]. HUVEC were from Lonza (Walkersville, MD) and were grown in EBM2 media with EGM-2 SingleQots supplements. Matrigel and invasion chamber were from BD Biosciences (New Bedford, MA). TUNEL assay kit was from Promega (Madison, WI). Carboxymethylcellulose (CMC), Harris hematoxylin and β-actin antibody were from Sigma (St. Louis, MO). DAB kit was from Vector Laboratories (Burlingame, CA). Streptavidin and PCNA antibody were from Dako (Carpinteria, CA). Antibodies for CD31, VEGF and nestin were from Abcam (Cambridge, MA). Antibodies for VEGFR1, VEGFR2, and HIF-1α [used for immunohistochemistry (IHC) analysis] as well as antibodies for Cdk2, Cdk4, Cdc2, cyclin D1, cyclin D3, cyclin B1, p21, p27, Skp 2, Cdc25A and Cdc25C and normal goat serum were from Santa Cruz Biotechnology (Santa Cruz, CA). p27 antibody was from Neomarkers (Fremont, CA) and p21 antibody was from Upstate (Charlottesville, VA). Antibodies recognizing the phosphorylated and/or total protein levels of VEGFR1, VEGFR2, Src, ERK1/2, Akt, Bad, mTOR, p70S6K, cleaved caspase 3, cleaved caspase 9, and goat anti-rabbit HRP-conjugated secondary antibodies were from Cell Signaling (Beverly, MA). ECL detection system and anti-mouse HRP-conjugated secondary antibody were from GE Healthcare (Buckinghamshire, UK). VEGF was from R & D (Minneapolis, MN). Silybin A, silybin B, isosilybin A and isosilybin B were isolated (purity >97%) from powdered extract of the fruits of *Silybum marianum* (L.) Gaertn. [obtained from Euromed, S.A. (Barcelona, Spain)]. The hybrid chromatographic/precipitative techniques and procedures for the gram scale purification of these flavonolignans are already reported previously [26].

### *In Vivo* Tumor Xenograft Study

Athymic (*nu/nu*) male nude mice were from the NCI (Frederick, MD). The treatment protocol was approved by the Institutional Animal Care and Use Committee of the University of Colorado Denver. About 3 × 10^6^ DU145 cells were suspended in 50 µL of serum free medium, mixed with 50 µL of matrigel, and injected s.c. in the right flank of each mouse. The day following xenograft implantation, mice were randomly divided into nine groups, and all treatments were done by oral gavage as: Group I mice (vehicle control group) with 200 µL of 0.5% CMC (w/v) in sterile water; Groups II and III mice with 50 and 100 mg/kg body weight dose of silybin A; Groups IV and V mice with 50 and 100 mg/kg body weight dose of silybin B; Groups VI and VII mice with 50 and 100 mg/kg body weight dose of isosilybin A; and Groups VIII and IX mice with 50 and 100 mg/kg body weight dose of isosilybin B, respectively. All these treatments (5 days/week) were given in 200 µL of 0.5% CMC. As all four compounds have same molecular weight (482.1), each dose-level was equimolar across these agents. Once tumor xenograft growth commenced, tumor sizes were measured twice weekly using digital caliper and tumor volume was calculated by the formula: 0.5236 L_1_(L_2_)^2^, where L_1_ is long diameter, and L_2_ is short diameter.

### IHC Analyses

Tumor samples were processed and immuno-stained following published methods [27], [28], [29]. Percentage of PCNA, TUNEL, cleaved caspase 3 and HIF-1α positive cells was calculated by counting the number of positive stained cells (brown stained) and the total number of cells at five arbitrarily selected fields from each tumor at 400x magnification. Microvessels stained with CD31 and nestin were quantified in 5 random microscopic (400x magnification) fields per tumor. VEGF, VEGFR1, VEGFR2, pAkt^ser473^, and Akt immunoreactivity was analyzed in 5 random areas for each tumor tissue and was scored as 0+ (no staining), 1+ (weak staining), 2+ (moderate staining), 3+ (strong staining), 4+ (very strong staining).

### *Ex Vivo* Capillary Formation Assay

Aortas isolated from mice were cleaned, cut into small fragments and placed on matrigel-covered wells and covered with another 100 µL matrigel. After these aortas were cultured for 24 h, the medium (complete HUVEC media) was replaced with or without flavonolignans (30 and 60 µM). Treatments were replaced after 48 h. After 6 days of total incubation, vessels sprouting from the aortas were photographed using Cannon Power Shot A640 camera on Zeiss inverted microscope.

### Tube Formation Assay

HUVEC (4 × 10^4^) were cultured in 1 mL EBM2 (supplemented with 0.5% FBS and 4 ng/mL VEGF) with various diastereoisomers (5–30 µM) on matrigel coated plates. After 9 h of incubation, tubular structure formation was quantified by calculating the tube length (at 100x) with Zeiss inverted microscope using Cannon Power Shot A640 camera and AxioVision Rel.4.7 software. In a related experiment, DU145 cells were treated with 30 µM dose of each flavonolignan for 72 h, and fresh media (0.5% FBS) was added and collected after 12 h (labeled as ‘conditioned media’). The conditioned media mixed with 0.5% FBS supplemented EBM2 media (75∶25 ratio) was then added to HUVEC and tube formation was studied as described above.

### Cell Viability Assay

HUVEC were treated with or without VEGF (10 ng/mL) and different concentrations of flavonolignans (5–30 µM). After the desired treatment, total cell number was determined using a hemocytometer.

### Transwell Invasion Assay

In this assay, the bottom chambers of Transwell were filled with EBM2 media containing 0.5% FBS supplemented with 4 ng/mL VEGF, and in the top chambers HUVEC (4 × 10^4^) were seeded in 500 µL EBM2 (0.5% FBS) plus 30 µM dose of each flavonolignan. After 10 h, invasive cells were quantified as described previously [30], [31]. Similar assay was also performed with DU145 cells plated in the bottom chamber (RPMI media with 0.5% serum) and HUVEC (EBM2 media with 0.5% serum) in the upper chamber. In two separate experiments, flavonolignans were added either in the upper chambers or in the lower chambers and invasiveness of HUVEC was studied in each case.

### Cell Cycle Distribution and Apoptosis

Cell cycle distribution (saponin/PI staining) and apoptosis (Annexin-PI staining) were analyzed by FACS [32], [33].

### Immunoblotting

HUVEC were treated with 30 µM dose of each flavonolignan with or without VEGF stimulation, lysates were prepared and analyzed by standard immunoblotting method as described earlier [33], [34]. α-tubulin and β-actin were used to confirm equal protein loading.

### Three Dimensional Spheroid Formation Assay

In 24 well plates, 100 µL of matrigel was added. Thereafter, 100 µl of RPMI-1640 and matrigel mixture (50∶50) containing ∼1×10^3^ DU145 cells was added. After 15 min, 1 mL RPMI-1640 medium with 10% FBS containing DMSO vehicle or 90 µM concentration of each flavonolignan was added in the well; treatments were replaced every 48 h for 2 weeks. At the end of the experiment, spheroid formation was counted under Zeiss inverted microscope at 100x.

### Statistical Analyses

Statistical analyses were performed using Sigma Stat software version 2.03 (Jandel Scientific, San Rafael, CA). The statistical significance of differences between control and treated-groups was determined by Student’s t test and p < 0.05 value was considered significant. One way ANOVA followed by Tukey’s test was used for multiple comparisons. The autoradiograms/bands were scanned, and mean density of bands (where mentioned) was determined using Adobe Photoshop 6.0 (Adobe Systems, San Jose, CA).

## Results

### Effect of Flavonolignans on PCA DU145 Xenograft Growth

In terms of anti-tumor efficacy, the oral administration of flavonolignans effectively inhibited the growth of DU145 xenografts in nude mice, and this was discernible from the third week onwards (Fig. 1A). At the end of the experiment (10 wks), silybin A treatment inhibited the tumor volume by 44 and 50% with 50 and 100 mg/kg body weight doses, respectively (p< 0.05) (Fig. 1A); while silybin B inhibited the tumor volume by 38 and 47% at same doses, respectively (p< 0.05) (Fig. 1A). In isosilybin A treated mice, tumor volume was inhibited by 44 and 50% (p< 0.05) (Fig. 1A), while in mice treated with isosilybin B the tumor volume was inhibited by 46 and 67% with 50 and 100 mg/kg body weight doses, respectively (p< 0.05) (Fig. 1A). Overall, isosilybin B was most effective in inhibiting the DU145 xenograft growth, followed by silybin A or isosilybin A and silybin B. Although, these differences in the biological effect of each diastereoisomer did not achieve statistical significance; these results were consistent with previously reported *in vitro* studies [21], [22].

At the time of necropsy, all animals were examined for gross pathology, and we did not observe any signs of abnormality in all the vital organs examined. Furthermore, the administration of these compounds through oral gavage did not cause any significant change in the diet consumption pattern or body weight gain of mice (data not shown). Also, we did not observe any adverse effect in terms of general behavior of animals, suggesting an overall safe nature of these compounds.

### Effect of Flavonolignans on Proliferation and Apoptosis in DU145 Xenografts

The IHC analysis of DU145 tumor samples showed that flavonolignans treatment significantly inhibits the immunostaining for PCNA (a biomarker for cell proliferation) (Fig. 1B), but increases the TUNEL and cleaved-caspase 3 positive cells (biomarkers for apoptosis) (Fig. 1C and 1D). Though statistically significant, the effect of flavonolignans on proliferation and apoptosis related biomarker was modest, and could not completely explain the observed slower xenograft growth and close to 50% growth inhibition with the flavonolignan treatment (Fig. 1A). Therefore, next we analyzed the tumor tissues for flavonolignans effect on angiogenesis, which is absolutely necessary for the tumors to grow beyond 1–2 mm size [3].

### Effect of Flavonolignans on Angiogenesis in DU145 Xenografts

To investigate whether these individual flavonolignans inhibited the xenograft growth by suppressing tumor angiogenesis, we stained the tumor section with CD31 (a biomarker for matured microvessels) and nestin (a biomarker for immature and newly formed microvessels). All four compounds inhibited the microvessel density, both mature and newly forming, but isosilybin B was relatively more effective in its inhibitory effect on nestin-positive microvessels (Fig. 2). VEGF is a potent pro-angiogenic factor [17], [35], and IHC analyses of tumor sections clearly showed that treatment with these diastereoisomers decreases both VEGF and VEGFR2 expression in tumor tissues (Fig. 2). Notably, except silybin A, VEGFR1 expression was also significantly decreased by these agents (Fig. 3). Overall, isosilybin B was relatively more potent in its efficacy on VEGF, VEGFR2 and VEGFR1 expression. HIF-1α is the master transcriptional factor that is stabilized under hypoxic conditions in growing tumors and controls tumor metabolism as well as expression of pro-angiogenic factors such as VEGF [36], [37], [38]. Although HIF-1α stabilization occurs under low oxygen conditions, its expression is also controlled by the serine/threonine kinase Akt [39]. Akt also plays an important role in multiple cellular processes, such as cell survival, apoptosis, migration and metabolism [40], [41]. IHC analyses of tumors showed that all four of the flavonolignans strongly decrease HIF-1α and pAkt^ser473^ levels, with marginal effect on total Akt expression only by isosilybin A and isosilybin B (Fig. 3).

### Effect of Flavonolignans on Angiogenesis in Non-target Organs

To confirm that the anti-angiogenic effects of these flavonolignans are specific to tumor tissues, we analyzed the non-target organs (liver, lung, and kidney) for CD31 expression to determine the microvessels density. There was no difference in the microvessel density in liver, lung and kidney between control and mice fed with pure flavonolignans (Fig. 4A, CD31 quantification data not shown). H & E analyses also showed that these diastereoisomers have no adverse effect on the histology of normal organs (Fig. 4B).

### Effect of Flavonolignans on Angiogenesis in *Ex Vivo* and *In Vitro* Assays

We further examined the anti-angiogenic activity of the flavonolignans in *ex vivo* capillary formation assay using mouse dorsal aortas. After six days of culture on matrigel under angiogenic conditions, we observed a significant number of vessels sprouting from the mouse aortas (marked by arrows), which was inhibited in a dose-dependent manner by flavonolignans treatment (Fig. 5A).

One of the important steps during neo-angiogenesis is the formation and merging of tubes produced by endothelial cells forming a complex network of vessels and capillaries [42], [43]. To understand the effect of flavonolignans on this biological event, we performed tube formation assay. As shown in Fig. 5B, bottom panel, all four compounds significantly inhibited the VEGF-induced tube length in HUVEC. As shown in the pictures (Fig. 5B, top panel), VEGF treatment induced the formation of tubular networks by HUVEC, which was disrupted by flavonolignan treatments.

### Effect of Flavonolignans on VEGF-induced Proliferation and Chemotactic Motility

VEGF plays an important role during neo-angiogenesis through its mitogenic and motogenic effect on endothelial cells [17], [35]. In our studies, VEGF treatment induced the HUVEC growth that was strongly inhibited by flavonolignans treatment (Fig. 6A). Such treatments also inhibited the chemotactic motility of HUVEC towards VEGF in the Transwell invasion assay (Fig. 6B). Importantly, isosilybin B was the least effective compared to the other diastereoisomers in terms of inhibitory effect on the chemotactic motility of HUVEC.

### Effect of Flavonolignans on Viability, Cell Cycle Progression and Apoptosis in HUVEC

To further elucidate the biological effect of these flavonolignans on endothelial cells, we analyzed viability, cell cycle and apoptosis in HUVEC. As shown in Fig. 7A, the four flavonolignans (5–30 µM) inhibited HUVEC viability in a dose- and time-dependent manner. Cell cycle analyses revealed that all the diastereoisomers induced G1 arrest after 24 h of treatment, but only silybin A, silybin B and isosilybin A also caused G2/M arrest (Fig. 7B). After 48 h of treatment, the noticeable effect of silybin A, silybin B and isosilybin A in HUVEC was the induction of G2/M arrest, which was missing with isosilybin B treatment (Fig. 7B). Isosilybin A treatment (at 30 µM) significantly induced S-phase arrest after 48 h of treatment, which could be linked to strong apoptotic death induced by isosilybin A (Fig. 7B).

We next examined the effect of these flavonolignans on various cell cycle regulatory molecules, namely cyclins, Cdks and Cdk inhibitors as well as their regulators Skp2 and phosphatases Cdc25. As shown in Fig. 7C, the four flavonolignans decreased the levels of Cdk2 and Cdk4 with marginal effect on Cdc2. These compounds also decreased the levels of cyclin D1, cyclin D3, cyclin A, and cyclin B1. Silybin A and isosilybin A moderately increased the p27 expression, but it was decreased by isosilybin B (Fig. 7C). On the contrary, p21 expression was decreased by silybin A, silybin B and isosilybin A but not by isosilybin B (Fig. 7C). However, all four diastereoisomers strongly decreased the expression of Skp2 and Cdc25C with no or moderate inhibitory effect on Cdc25A level (Fig. 7C). These results suggested that these four diastereoisomers have few similar (but differing in the extent) and few contrasting effects on the expression of cell cycle regulatory molecules.

We then examined the effect of the pure flavonolignans (at 30 µM) on apoptosis after 36 h of treatment in HUVEC. As shown in Fig. 7D, isosilybin B was the least efficacious in terms of inducing apoptosis related morphological features (detachment and rounding), signaling molecules involved in apoptosis (cPARP, cleaved caspase 3 and 9) and percentage of apoptotic cell population in HUVEC. Conversely, silybin A, silybin B and isosilybin A strongly induced apoptotic death in HUVEC (Fig. 7D).

### Effect of Flavonolignans on VEGF-induced Signaling in HUVEC

Next, we examined flavonolignans effect on the VEGF-induced signaling cascade that controls proliferation, motility and tube formation in endothelial cells [35]. VEGF treatment strongly increased the VEGFR2 phosphorylation at Ser1175 site, a reliable marker for its activity, which was inhibited by pre-treatment with flavonolignans (Fig. 8). Similarly, VEGF increased the phosphorylation of VEGFR1, Src, ERK1/2, Akt, BAD, mTOR, and p70S6K. As shown in Fig. 8, except ERK1/2 and mTOR, phosphorylation at other sites was inhibited by these compounds albeit to different extent. Compared to its other diastereoisomers, isosilybin B was the least effective in terms of effect on the VEGF-induced signaling molecules in HUVEC.

### Differential Sensitivity of Flavonolignans Towards PCA and Endothelial Cells

Earlier findings [20], [21], [22], [24] and the present study suggested that among the four pure flavonolignans, isosilybin B is the most effective in terms of efficacy against PCA cells but, surprisingly, it has the least efficacy in terms of its effect on HUVEC (inhibition of chemotactic motility or apoptosis induction) (Figs. 6 and 7). Therefore, we performed a series of experiments to elucidate the relative efficacy of these four diastereoisomers against PCA cells vis-à-vis endothelial cells. First, we analyzed their effect on the three dimensional growth of DU145 cells in matrigel. Treatment with these compounds strongly inhibited the number and size of spheroid formed by DU145 cells with isosilybin B being most effective (Fig. 9A). Next, we performed co-culture studies using Transwell chambers. We plated HUVEC in the upper chamber, while DU145 cells were cultured in the lower chamber. In two separate experiments, HUVEC or DU145 cells were treated with pure flavonolignans, and thereafter, HUVEC invasion through matrigel was studied in each case. As shown in Fig. 9B, isosilybin B was the most effective in decreasing the HUVEC migration when DU145 cells were treated but was least effective when HUVEC were treated.

To follow this, we treated DU145 cells with these diastereoisomers (30 µM) and collected the conditioned media. The pro-angiogenic potential of the conditioned media was analyzed in a tube formation assay using HUVEC. As shown in Fig. 9C, conditioned media from DU145 cells increased the tube length as well as tubular network formed by HUVEC. Conditioned media from the flavonolignan treated DU145 cells significantly decreased the tube length as well as tubular network (Fig. 9C). In this assay too, isosilybin B was the most effective diastereoisomer (Fig. 9C). On the contrary, silybin A was more effective when HUVEC were treated directly with these diastereoisomers (Fig. 5B). Together these studies further confirmed the differential sensitivity of these diastereoisomers towards PCA and endothelial cells.

## Discussion

Neo-angiogenesis is the critical step in the development and progression of most of the human cancers. Beyond the critical size of 1–2 mm, oxygen and nutrients have difficulty in diffusing to the core cells of the tumor, causing a state of cellular hypoxia. Under hypoxic conditions, cancer cells secrete several pro-angiogenic factors such as VEGF and bFGF, which cause recruitment of endothelial cells from the neighboring blood vessels [6], [44]. But the continuous and excessive presence of pro-angiogenic stimuli in the tumor microenvironment interferes with the normal maturation of vessel network, and as a result, vessels in the tumor area show abnormal morphology and physiology. Hence, it is possible to specifically target abnormal tumor angiogenesis by inhibiting endothelial cell recruitment as well as their proliferation in the tumor microenvironment. Notably, the results from the present study clearly suggest the strong efficacy of four pure flavonolignans from Milk Thistle extract, on these aspects, which signifies their angiopreventive efficacy against PCA. These findings are aslo transnationally noteworthy, as currently, anti-angiogenic strategies are extensively pursued towards preventing the progression of diagnosed pre-malignant tumors or for the therapeutic regression of the already advanced disease [16], [45].

Milk thistle has been used for centuries to treat chronic liver disease, and to protect the liver against toxins [46]. In recent years, Milk thistle research use has grown significantly with close to two dozen clinical trials underway or already completed evaluating its efficacy against variety of diseases including chronic hepatitis, diabetes, asthma, mushroom poisoning and various cancers (these studies are listed at ClinicalTrials.gov). In the past, there have been numerous efforts in isolating and purifying the individual constituents in Milk thistle to better exploit its clinical usefulness. Members of our team were the first to purify and elucidate seven distinct flavonolignans from Milk Thistle extract, namely: silybin A, silybin B, isosilybin A, isosilybin B, silydianin, silychristin, and isosilychristin, and one flavonoid, taxifolin [47]. Their biological effects as pure compounds were assessed on several anti-proliferative end points in human PCA cell lines, where isosilybin B ranked as the most potent flavonolignan for nearly all the end points, including the inhibition of cell growth, clonogenic potential, PSA and androgen receptor levels, and topo IIα promoter activity [20], [21], [22], [24]. Despite these advances in the Milk Thistle research, the *in vivo* biological efficacy and related toxicity of these pure flavonolignans remained unknown because of insufficient compound quantities. In particular, isosilybin B was the most challenging of the major diastereoisomer to purify, due to its limited abundance in the natural extract [21] and its relatively long retention time in most reverse-phase HPLC systems. The pre-clinical or future clinical use of these pure compounds needed advancement in their isolation; therefore, we developed a hybrid chromatographic/precipitative technique for gram scale purification of flavonolignan diastereoisomers from Milk Thistle extract [26]. We believe that this is a major advancement towards the future translational significance of these pure flavonolignans.

The results from the present study adequately proved that the pure diastereoisomers from Milk Thistle have strong angiopreventive efficacy through targeting the pro-angiogenic signaling in PCA cells as well as in endothelial cells; the important component of PCA microenvironment (Fig. 10). Furthermore, based upon the overall *in vitro* and *in vivo* analyses, it is also clear that the comparative efficacy of isosilybin B occupies two opposite ends in terms of its effect on PCA cells and endothelial cells. Specifically, it is the lead agent in terms of its efficacy against PCA cells but it is the least effective agent against endothelial cells. On the contrary, silybin A appears to be the most promising agent in terms of its effects on endothelial cells. Therefore, it is prudent to suggest that a defined mixture of silybin A and isosilybin B should be tested, particularly *in vivo*, with an expectation of these two compounds acting in concert to exert maximum benefits via targeting both the tumor and the tumor microenvironment components.

In summary, the present study, for the first time, report the anti-cancer efficacy of four flavonolignans from Milk Thistle extract in an *in vivo* system of advanced stage human PCA. These results further suggest the stereochemistry based differential efficacy of these flavonolignans towards cancer and endothelial cells. Furthermore, these results confirm the non-toxicity as well as PCA specific anti-angiogenic effects of the pure diastereoisomers, which suggest their usefulness in PCA angioprevention.
